# Effects of carbon nanotube addition on the microstructures, martensitic transformation, and internal friction of Cu–Al–Ni shape-memory alloys

**DOI:** 10.1038/s41598-023-48516-4

**Published:** 2023-12-01

**Authors:** Mozhgan Gholami-Kermanshahi, Yuan-Chien Hsiao, Günther Lange, Shih-Hang Chang

**Affiliations:** 1 Group for Metallic and Composite Materials, TU Ilmenau, Gustav-Kirchhoff-Str. 6, 98693 Ilmenau, Germany; 2 Department of Chemical and Materials Engineering, National I-Lan University, I-Lan 260, Taiwan

**Keywords:** Engineering, Materials science

## Abstract

In this study, we analyze the influences of carbon nanotube (CNT) addition on the martensite transformation and internal friction of Cu–Al–Ni shape-memory alloys (SMAs). X-ray diffraction and differential scanning calorimetry results demonstrate that Cu–13.5Al–4Ni–*x*CNT (*x* = 0, 0.2, 0.4, 0.6, and 0.8 wt%) SMA/CNT composites exhibit a ${\upbeta }_{1}({\mathrm{DO}}_{3})\rightleftarrows {\upbeta }_{1}^{\mathrm{^{\prime}}}(18\mathrm{R})$ martensitic transformation. The martensitic transformation temperatures and transformation enthalpies of the martensitic transformation peaks for the Cu–13.5Al–4Ni–*x*CNT (*x* = 0–0.8 wt%) composites gradually decrease with the increase in the amount of CNT addition. Compared to the Cu–13.5Al–4Ni SMA, the Cu–13.5Al–4Ni–*x*CNT (*x* = 0.2–0.8 wt%) SMA/CNT composites exhibit significant improvements in the amount of dissipation of energy (storage modulus (${E}^{\prime}))$ and mechanical strength. However, the tan δ of the internal friction peak gradually decreases with the increase in the CNT content above 0.6 wt%. The reduction in tan *δ* is attributed to the decrease in the magnitude of the austenite-to-martensite transformation and precipitation of γ_2_ (Cu_9_Al_4_) phase particles, which impede the interface motion in between the parent/martensitic phase and martensitic phase.

## Introduction

Shape memory alloys (SMAs), such as Ni–Ti-based, Cu-based, and Fe-based alloys are functional materials whose properties trace back to a reversible martensitic transformation^1^. Martensitic transformation (MT) is induced by changing the temperature or under the influence of an external stress^2^. Both temperature-induced martensite and stress-induced martensite are the characteristic thermomechanical properties in the SMAs, which trigger shape memory effect and superelasticity. These two physical behaviors make SMAs viable materials to address mechanical vibrations in structural applications^3,4^.

The aggressive demand for high-performance damping materials is crucial to a wide range of applications for civil, mechanical, and aerospace systems due to their capability to reduce vibration and acoustic noise^1,5–7^. High-damping materials possess the ability to suppress undesirable mechanical vibrations and acoustic wave propagation by energy dissipation^8^. The damping capacity of SMAs stems from the wasted energy due to the movement of the variant interfaces (such as parent-martensite habit planes, martensite-martensite interfaces, twin planes, and grain boundaries), the dislocations relaxation, and the interaction between dislocations/dislocation and dislocations/point defects^9–11^. Consequently, the amount of martensite and the density of the different phase interfaces play a significant role in the damping properties. The refinement of the size of the grain structure and martensite phase is the preferred solution to increase the interfacial density^12^.

Cu–Al-based damping alloys have gained the interest of researchers in recent years due to their low cost, high transition temperatures, the simplicity of manufacture, and the unique thermoelastic martensitic structure^9,13^. Recently Cu–Al–Ni SMAs are acting as a prominent class of Cu-based SMAs for developing materials for various applications such as high-damping capacity materials, sensors, and actuators due to their high thermal stability rather than other Cu-based SMAs^14^. On the other hand, it is necessary to notice that some high-damping materials exhibit poor mechanical properties^15^.

A great deal of efforts made by researchers to enhance damping and mechanical properties of Cu–Al-based alloys for obtaining a structurally integrated SMA are summarized in three aspects: heat treatment, the addition of alloying elements and nano-reinforcement^9,16^. Furthermore, despite alloying elements and heat treatment, which play a significant role in enhancing mechanical and damping properties, nanomaterials as a reinforcing phase are also attracting more attention from researchers for obtaining materials with more desirable properties. Nanoparticles with a range of 1–100 nm and a large surface area to the volume ratios show full potential in enhancing the functional properties of metals by impeding the dislocation motion^17,18^.

Despite the significant improvement of mechanical properties, the nanoparticles have received intensive attention to improving the damping properties. The achieved improvement of damping by nanomaterials is mainly due to the energy dissipation through interfacial sliding and friction between the matrix and the nanoreinforcement^19,20^. Furthermore, the existence of high-density dislocation accumulations around the nano-reinforced phase gives rise to a drastic increase in damping capacity. A varying thermal expansion coefficient between the matrix and nano-reinforced particles interprets the occurrence of a dislocation-damping mechanism. When moving dislocations become pinned by nanoparticles or other impediments, they act as elastic vibrating strings, which lead to energy dissipation^20,21^.

Many studies have shown that nanoparticles such as Al_2_O_3_, ZrO_2_, and Y_2_O_3_ are necessary for industrial applications that demand excellent mechanical properties and damping performance of metal-based engineering materials^22,23^. This performance improvement of parent material is mainly due to the percentage increase of finer grains, grain-boundary strengthening, solid solution strengthening, and dispersion strengthening. The improvement in the mechanical and functional properties of the modified Cu-based alloys with the addition of nano-reinforcement derives from the unique feature of their nanoscale structure with high strength and diffusivity. The addition of nanoparticles to Cu–Al-based alloys results in rational control of the microstructure from the perspective of refining the grain size compared to alloying and heat treatment processes. The effect of fine-grained strengthening and interfacial damping significantly improves the comprehensive performance of the Cu–Al-based alloys^9^.

It is noteworthy that graphene and carbon nanotubes (CNTs) indicate the potential to improve the strength and damping characteristics of metal-based materials^24,25^. As one of the promising engineering damping materials, CNTs-based metals have attracted widespread attention owing to their optimum modulus, high strength, thermostability, and high thermal conductivity^26^. Saud et.al.^27^ indicated that adding the CNTs to Cu–Al–Ni–SMAs significantly enhances the mechanical properties and shape memory effects using controlling the microstructure. Furthermore, CNTs contribute to damping capacity improvement due to their extremely large specific surface area (interfacial surface area per unit of mass) and low density. The damping capacity improvement of CNTs-based materials is primarily defined as a function of the energy dissipation due to the interfacial slip and friction between the walls of CNTs and the CNTs-matrix^24^.

It is necessary to design Cu-based SMAs modified by CNTs with comprehensive mechanical and damping properties to meet the practical requirements for engineering materials. The introduction clarified a brief overview of substantial improvements in the mechanical and damping properties of materials by nano-reinforcements. However, it is rather lacking in studying the mechanisms and effects of nano-particles on the damping performance of Cu–Al-based alloys. This study experimentally investigates the effect of adding various contents of CNTs on the damping performance of Cu–13.5Al–4Ni SMA.

## Experimental

This study is focused on the influence of CNT addition on the damping performances of Cu–13.5Al–4Ni–*x*CNT (*x* = 0–0.8 wt%) composites. Ingots of Cu–13.5Al–4Ni–*x*CNT composites were prepared using high-purity (99.9%) copper, aluminum, and nickel with various amounts of multiwall CNT powders (diameter of 5–20 nm; length > 1 µm; purity > 98%) by a conventional vacuum arc furnace. The Cu–Al–Ni SMA/CNT composite ingots were homogenized at 900 °C for 30 min, and then quenched in ice water to form the martensite.

The quenched Cu–Al–Ni SMA/CNT composite ingots were cut into bulks with dimensions of approximately 30.0 mm × 5.0 mm × 2.5 mm using a low-speed diamond saw (IsoMet LS, Buehler) for dynamic mechanical analysis (DMA), X-ray diffraction (XRD), scanning electron microscopy (SEM), and hardness measurements. The quenched Cu–Al–Ni SMA/CNT composite ingots were also cut into segments of approximately 30 mg for differential scanning calorimetry (DSC) tests. The crystallographic characteristics of the Cu–Al–Ni SMA/CNT composites were analyzed by a Rigaku Ultima IV XRD instrument with Cu K_α_ radiation (*λ* = 0.154 nm) at room temperature. DSC (TA Q10) was performed under a constant heating and cooling rate of 10 °C/min to estimate the phase transformation temperatures and transformation enthalpy (Δ*H*). Microstructures and chemical compositions of the specimens were investigated by SEM (Tescan 5136MM) equipped with energy-dispersive X-ray spectroscopy (EDS) (X-Act, Oxford). The damping capacity (tan δ) and storage modulus were determined using a DMA (TA 2980) equipment with a single cantilever clamp and liquid-nitrogen cooling apparatus. DMA is a technique used to characterize materials by applying a sinusoidal deformation to a sample. In a DMA test, the storage modulus (${E}^{\prime}$) and loss modulus (${E}^{{\prime}{\prime}})$ indicate the stored energy and dissipation of energy in the specimen, respectively. The internal friction (tan $\delta$) is defined as the ratio between the loss modulus and storage modulus ($\mathrm{tan}\delta =\frac{{E}^{{\prime}{\prime}}}{{E}^{\prime}}$). Therefore, when tan δ is smaller than 1, the complex modulus (${E}^{*}={E}^{\prime}+i{E}^{{\prime}{\prime}})$ is considered almost equal to the storage modulus because the loss modulus is negligible. The tan δ and storage moduli of the Cu–Al–Ni SMA/CNT composites in this study were measured by DMA at a temperature rate of 3 °C/min, frequency of 1 Hz, and strain amplitude *ɛ* of 1.0 × 10^−4^. The hardnesses of the specimens were determined with a Vickers hardness tester (Wilson Instruments, 402 MVD) using a load of 300* g* and average dwell time of 10 s.

## Results and discussion

### *Crystallographic characterization of Cu*–*13.5Al*–*4Ni*–*xCNT composites*

**Figure 1:**
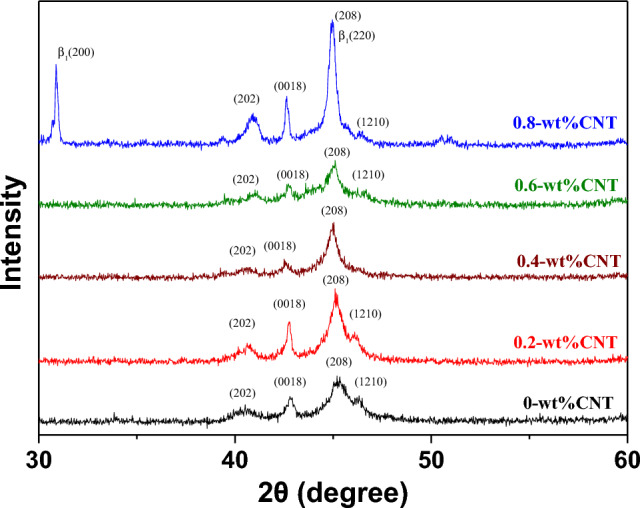
XRD results of the Cu–13.5Al–4Ni–xCNT (x = 0, 0.2, 0.4, 0.6, and 0.8 wt%) SMA/CNT composites.

Figure 1 compares XRD patterns of Cu–13.5Al–4Ni–*x*CNT (*x* = 0, 0.2, 0.4, 0.6, and 0.8 wt%) SMA/CNT composites. As shown in Fig. 1, each specimen exhibits diffraction peaks at 2*θ* = 40.52°, 42.80°, 45.34°, and 46.32°, which correspond to the (202), (0018), (208), and (1210) diffraction planes of the ${\upbeta }_{1}{\prime}$ martensite phase with a 18R structure at room temperature, respectively. At the CNT concentration of 0.8 wt%, the Cu–13.5Al–4Ni–0.8CNT composite exhibited not only the diffraction peaks of the ${\upbeta }_{1}{\prime}\left(18\mathrm{R}\right)$ martensite phase, but also the diffraction peaks corresponding to the (200) and (220) diffraction planes of $\upbeta {(DO}_{3})$. This indicates that the ${\upbeta }_{1}{\prime}\left(18\mathrm{R}\right)$ martensite and $\upbeta {(DO}_{3})$ parent phase coexist in the Cu–13.5Al–4Ni–0.8CNT composite at room temperature. The decreasing intensity of the martensite peaks reflects the reduction in the content of the austenite-to-martensite phase transformation, where a higher peak intensity shows a higher martensitic phase transformation^28^. The XRD results confirmed that, due to the low addition amount of CNTs and chemically stable properties, no diffraction peaks for CNTs were detected, because CNTs did not react with the other elements in the matrix, and therefore no new phase containing C was formed^29^. The XRD results also reveal that the polycrystalline Cu–13.5Al–4Ni SMA and Cu13.5Al–4Ni–*x*CNT contain a single ${\upbeta }_{1}{\prime}\left(18\mathrm{R}\right)$ martensite phase. The addition of CNTs did not influence the type of martensite phase. However, the increase in the CNT amount retarded the martensite transformation up to room temperature.

### *MT of Cu*–*13.5Al*–*4Ni*–*xCNT composites*

**Figure 2:**
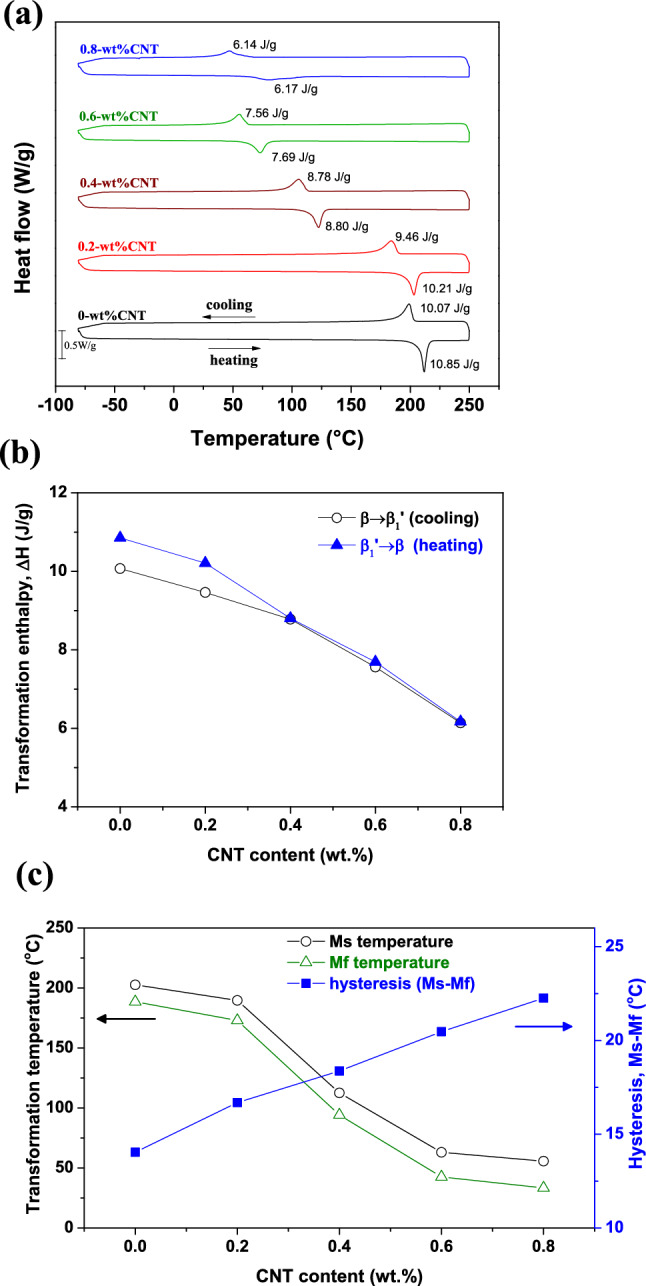
(**a**) DSC curves of the Cu–13.5Al–4Ni–*x*CNT (*x* = 0, 0.2, 0.4, 0.6, and 0.8 wt%) SMA/CNT composites. (**b**) Evolution of the transformation enthalpy determined by the DSC curve as a function of the CNT content. (**c**) Evolution of the Ms, Mf temperatures and hysteresis (Ms–Mf) determined by the DSC curve as a function of the CNT content.

Figure 2a shows DSC curves of the Cu–13.5Al–4Ni–*x*CNT (*x* = 0, 0.2, 0.4, 0.6, and 0.8 wt%) SMA/CNT composites obtained with a heating/cooling rate of 10 °C/min. Figure 2a reveals that only one exothermic peak, that of $\upbeta {(DO}_{3})$ → ${\upbeta }_{1}{\prime}\left(18\mathrm{R}\right)$, appeared during cooling, and one endothermic peak, that of ${\upbeta }_{1}{\prime}\left(18\mathrm{R}\right)$ → $\upbeta {(DO}_{3})$, appeared during heating in the DSC curves. Therefore, the martensite phase forms in the Cu–13.5Al–4Ni–*x*CNT SMA/CNT composite under a typical one-step $\upbeta {(DO}_{3})$ $\leftrightarrow$ ${\upbeta }_{1}{\prime}\left(18\mathrm{R}\right)$ MT^30^. The addition of CNT does not affect the one-step MT in the Cu–13.5Al–4Ni SMA, whereas the peak temperature of the $\upbeta {(DO}_{3})$ $\leftrightarrow {\upbeta }_{1}{\prime} \left(18\mathrm{R}\right)$ MT decreases as the CNT content increases. This is because the CNTs in the Cu–13.5Al–4Ni–xCNT composite impeded the movements of the interface between parent and martensite phases during transformation. Similar results are also reported by Chen et al.^31^ and Zhao et al.^32^.

Figure 2b shows the MT enthalpies ($\Delta H)$ determined by Fig. 2a as a function of the CNT content. Figure 2b shows that the MT enthalpies ($\Delta H)$ for $\upbeta {(DO}_{3})$ → ${\upbeta }_{1}{\prime}\left(18\mathrm{R}\right)$ and ${\upbeta }_{1}{\prime}\left(18\mathrm{R}\right)$ → $\upbeta {(DO}_{3})$ peaks gradually decrease with the increase in the CNT content in the Cu–13.5Al–4Ni–*x*CNT SMA/CNT composites. The reduced transformation enthalpies ($\Delta H)$ indicate a reduction in the magnitude of the austenite-to-martensite transformation where the Cu–13.5Al–4Ni–*x*CNT SMA/CNT composites with a higher $\Delta H$ contain a higher proportion of the martensite phase^10^. The DSC curves reveal an apparent delay in MT with the increase in the CNT content. The CNT addition effectively influenced the MT temperature, and shifted it to around room temperature. After the addition of CNT particles to the Cu–13.5Al–4Ni SMAs, the temperature range in which martensite is stable is decreased, and the austenite is stable in a wider range of temperature. Figure 2c shows the martensite start (Ms) temperature, martensite finish (Mf) temperature, and the hysteresis of the transformation temperatures (Ms–Mf) determined by the DSC curve as a function of the CNT content. Figure 2c reveals that both the Ms and Mf temperatures of the Cu–13.5Al–4Ni–*x*CNT SMA/CNT composites decrease with the increase of the CNT content, whereas the hysteresis (Ms–Mf) becomes wider at the same time. This is because the CNT and precipitated second phase typically inhibit the martensitic transformation of SMAs, causing a high energy dissipation during transformation^27,33^.

### *Microstructures of Cu*–*13.5Al*–*4Ni*–*xCNT composites*

**Figure 3:**
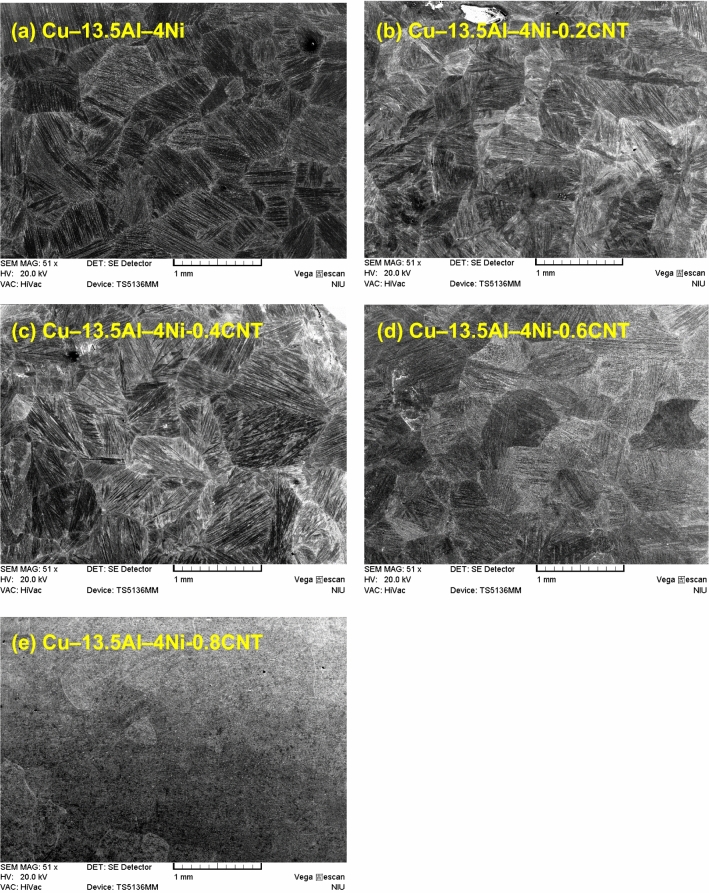
SEM images (50 ×) of (**a**) Cu–13.5Al–4Ni, (**b**) Cu–13.5Al–4Ni–0.2CNT, (**c**) Cu–13.5Al–4Ni–0.4CNT, (**d**) Cu–13.5Al–4Ni–0.6CNT, and (**e**) Cu–13.5Al–4Ni–0.8CNT.

**Figure 4:**
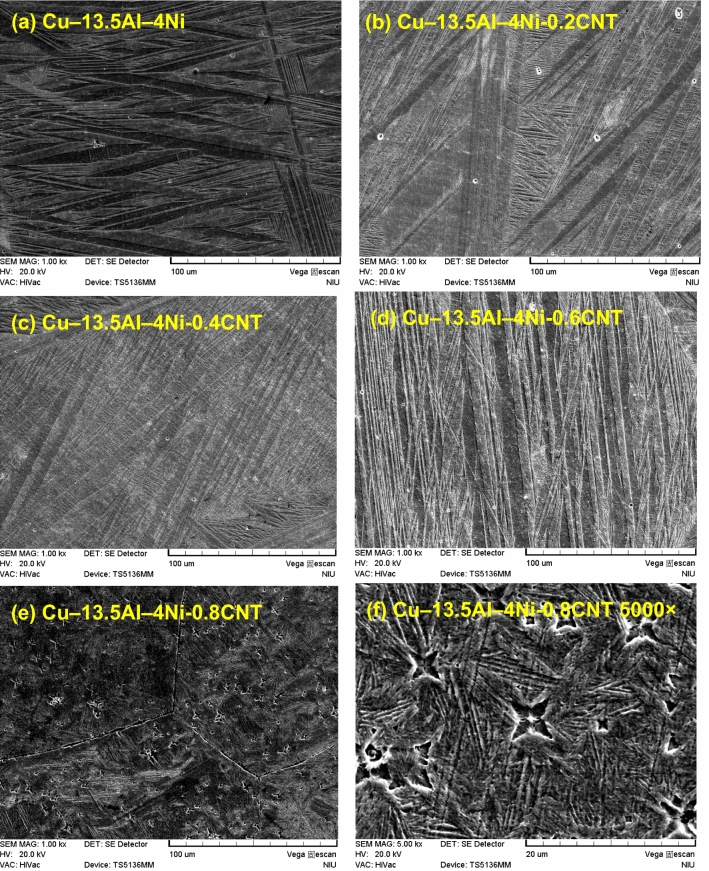
SEM images (1000 ×) of (**a**) Cu–13.5Al–4Ni, (**b**) Cu–13.5Al–4Ni–0.2CNT, (**c**) Cu–13.5Al–4Ni–0.4CNT, (**d**) Cu–13.5Al–4Ni–0.6CNT, and (**e**) Cu–13.5Al–4Ni–0.8CNT composites. (**f**) SEM image of the Cu–13.5Al–4Ni–0.8CNT SMA/CNT composite at a magnification of 5000 × .

Figure 3(a–e) show the microstructures of the Cu–13.5Al–4Ni–*x*CNT (*x* = 0, 0.2, 0.4, 0.6, and 0.8 wt%) SMA/CNT composites, respectively, determined by SEM. As shown in Fig. 3, the grain size of the Cu–13.5Al–4Ni–*x*CNT SMA/CNT composites is almost constant with the increase in the CNT content, because the CNTs can pin the grain boundaries and inhibit the grain growth^29^. Figure 4(a–e) also show the microstructures of the Cu–13.5Al–4Ni–*x*CNT (*x* = 0, 0.2, 0.4, 0.6, and 0.8 wt%) SMA/CNT composites, respectively, at a larger magnification (1000 ×). As shown in Fig. 4a, both lath martensitic structure ${\gamma }_{1}{\prime}$ and typical zig–zag self-accommodating group of ${\beta }_{1}{\prime}$ martensite variants are obtained in the Cu–13.5Al–4Ni SMA. Although two martensitic structures of ${\beta }_{1}{\prime}$ and ${\gamma }_{1}{\prime}$ are observed in the SEM image, only the ${\beta }_{1}{\prime}$ martensitic structure could be obtained in the XRD pattern in Fig. 1. This originated from the small quantity of the ${\gamma }_{1}{\prime}$ martensite phase in the structure. The ${\gamma }_{1}{\prime}$ phase could be recognized through XRD patterns when the ${\gamma }_{1}{\prime}$ content is larger than 5 wt%. Therefore, in Cu–13.5Al–4Ni, the martensitic structure was detected to be primarily ${\beta }_{1}{\prime}$, which leads to the characteristics of the shape-memory effects^30^.

As shown in Fig. 4(b–e), the addition of CNTs resulted in the formation of only ${\upbeta }_{1}{\prime}\left(18\mathrm{R}\right)$ martensitic phase in the Cu–13.5Al–4Ni–*x*CNT SMA/CNT composites. The martensitic structure formed after solution annealing (betatization) at 900 °C and quenching in the ice water. After a rapid quenching below the martensite start temperature, martensite plates with different orientations lay instantaneously in the original austenite grains. The structure of the thermoelastic martensite is characterized by a combination of many variants. The martensite variant distributions are parallel to each other. The parallel microstructure induces a high mobility in the martensitic structure during loading. Notably, the thermoelastic martensitic phase develops its potentially high damping capacity from the hysteretic mobility of martensite variant interfaces. A microstructure with more martensite variant interfaces exhibits a higher damping capacity^34^. The microstructure of the martensite contains self-accommodated needle-like plates. In some regions, the V-shaped martensite appeared. The microstructure observations of all Cu–13.5Al–4Ni–*x*CNT SMA/CNT composites indicate that the morphology of the resulting microstructure is a typical self-accommodating zig–zag morphology, which is characteristic of martensite in Cu-based SMAs^35^.

As shown in Fig. 4e, abundant precipitates exist in the Cu–13.5Al–4Ni–0.8CNT SMA/CNT composite. Figure 4f presents an SEM image of the same Cu–13.5Al–4Ni–0.8CNT SMA/CNT composite at a larger magnification (5000 ×), which shows that these precipitates possessed a crisscross structure. These precipitates are referred to as γ_2_(Cu_9_Al_4_) phase^36^, which considerably affects the phase transformation in the Cu–13.5Al–4Ni–*x*CNT SMA/CNT composites. The γ_2_ precipitates can only be obtained in the Cu–13.5Al–4Ni–0.8CNT SMA/CNT composite. This is because a large concentration of added CNTs could serve as heterogeneous nucleation sites in composite to generate the precipitation of second phases.

**Figure 5:**
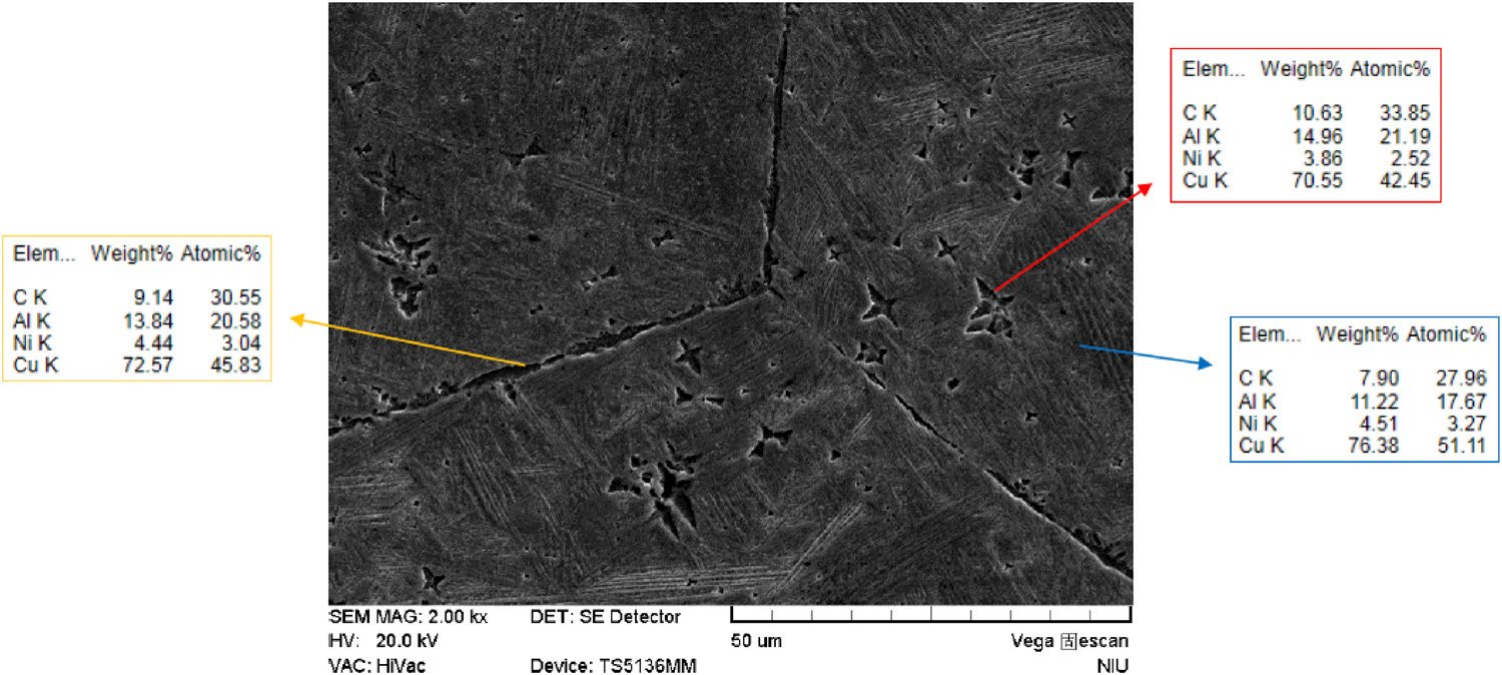
EDS results of the Cu–13.5Al–4Ni–0.8CNT SMA/CNT composite.

Figure 5 shows a detailed EDS analysis of the distribution of the CNTs and alloying elements in the matrix. The chemical analysis reveals an almost uniform dispersion of the CNTs in the matrix material. However, the tendency of agglomeration of the CNTs on the grain boundaries and crisscross γ_2_(Cu_9_Al_4_) phase is stronger than that in the matrix.

### *Hardness of the Cu*–*13.5Al*–*4Ni*–*xCNT composites*

**Figure 6:**
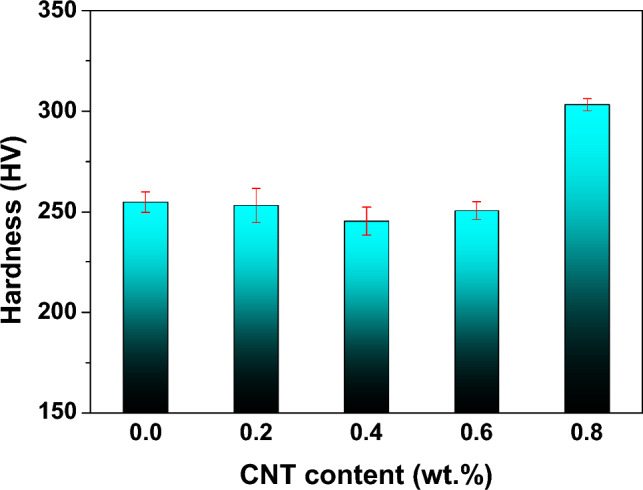
Hardness of the Cu–13.5Al–4Ni–*x*CNT (*x* = 0, 0.2, 0.4, 0.6, and 0.8 wt%) SMA/CNT composites as a function of the CNT content.

The hardness, strongly related to the strength of the material, is considered an indicative parameter of the mechanical properties of SMAs^37^. Figure 6 presents the effect of the addition of CNTs on the hardness of the Cu–13.5Al–4Ni–*x*CNT (*x* = 0, 0.2, 0.4, 0.6, and 0.8 wt%) SMA/CNT composites. As shown in Fig. 6, the hardness of the Cu–13.5Al–4Ni alloy without CNT addition was approximately 254.9 ± 5.0 HV. The hardnesses of the Cu–13.5Al–4Ni–0.2CNT, Cu–13.5Al–4Ni–0.4CNT, and Cu–13.5Al–4Ni–0.6CNT composites were 253.3 ± 8.5, 245.5 ± 6.9, and 250.6 ± 4.4 HV, respectively. This indicates that the addition of CNTs did not significantly affect the hardness of the Cu–13.5Al–4Ni SMA when the CNT content was below 0.6 wt%. Xue et al.^38^ reported that CNT-reinforced Cu matrix composites could induce a large area of interfaces between CNTs and Cu matrix, which increases the yield stress and tensile strength in Cu–CNT composites. The development of the tensile strength with more CNTs results from the larger interfacial area between CNTs and Cu matrix, which act as a bridge to load transfer during tensile testing. However, the hardness of the Cu–13.5Al–4Ni SMA was almost the same in this study when the CNT addition was below 0.6 wt%, likely because the content of the CNTs was considerably smaller than those reported by Xue et al.^38^ (Cu–5-vol.% CNTs).

Nevertheless, Fig. 6 shows that the hardness of the Cu–13.5Al–4Ni–0.8CNT composite is approximately 303.2 ± 4.4 HV, considerably higher than those of the other Cu–13.5Al–4Ni–*x*CNT composites. The increase in hardness of the Cu–13.5Al–4Ni–0.8CNT composite could be attributed to the precipitation of γ_2_ (Cu_9_Al_4_) phase particles when the CNT addition was above 0.8 wt%, as demonstrated in Fig. 4, which led to dispersion strengthening. This type of microstructure is beneficial for the improvement in strength through the effect of dispersion strengthening^39^. Similar results were reported by Murugesan et al.^40^, where the uniform distribution of CNTs within Al/Cu–CNT composites and formation of theta-Al_2_Cu strengthening precipitates led to a strong interfacial bonding and better load transfer between the matrix and reinforcement, which resulted in an enhancement in hardness.

### *Damping capacities of Cu*–*13.5Al*–*4Ni*–*xCNT composites*

**Figure 7:**
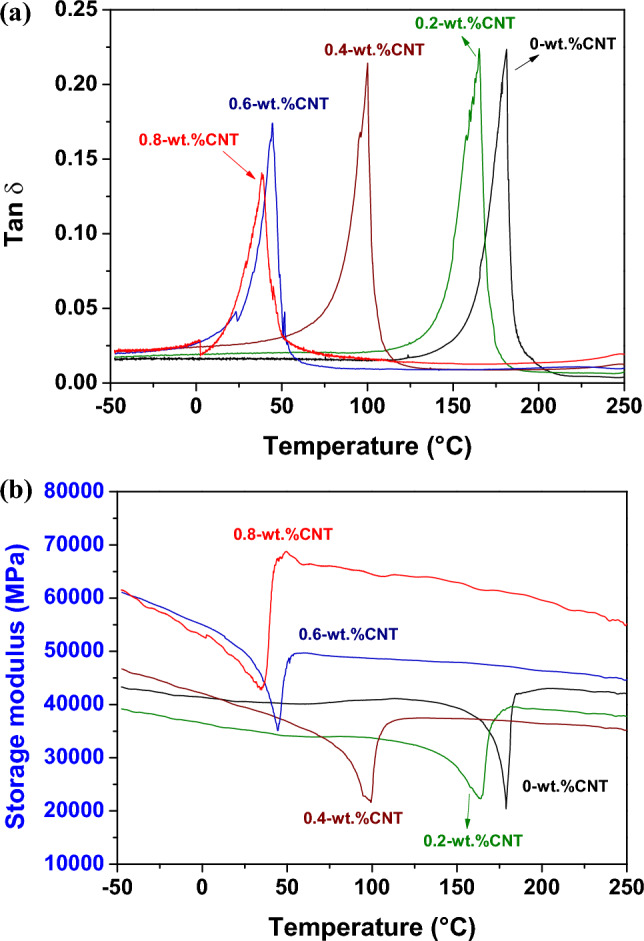
(**a**) Tan *δ* and (**b**) storage modulus curves of the Cu–13.5Al–4Ni–*x*CNT (*x* = 0, 0.2, 0.4, 0.6, and 0.8 wt%) SMA/CNT composite.

**Figure 8:**
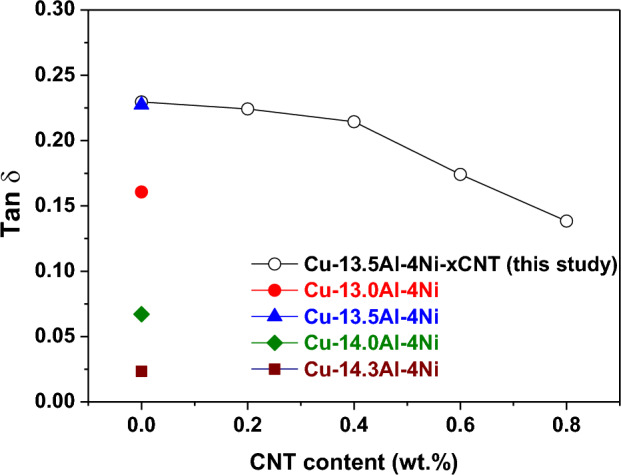
Tan *δ* values of the IF peaks of Cu–13.5Al–4Ni–*x*CNT (*x* = 0, 0.2, 0.4, 0.6, and 0.8 wt%) SMA/CNT composites determined from Fig. 7a. Tan *δ* values of the IF peaks of typical Cu–Al–Ni SMAs^36^ are also presented for comparison.

Figure 7a and b show tan δ and storage modulus curves of the Cu–13.5Al–4Ni–*x*CNT (*x* = 0, 0.2, 0.4, 0.6, and 0.8 wt%) SMA/CNT composites, respectively. Only the cooling curves are presented in Fig. 7 for clarity. As shown in Fig. 7a, each Cu–13.5Al–4Ni–*x*CNT specimen exhibits a $\upbeta {(DO}_{3})$ → ${\upbeta }_{1}{\prime}\left(18\mathrm{R}\right)$ internal friction peak in the tan δ curve, which corresponds to the observed peak in the DSC curve in Fig. 2. The peak temperatures of each specimen measured by DSC and DMA tests exhibit a small shift because of the different cooling rates and specimen sizes^41^. The Cu–13.5Al–4Ni–*x*CNT composites containing 0, 0.2, and 0.4-wt% CNTs exhibit a significant internal friction peak with tan δ values above 0.2. However, the tan δ value of the internal friction peak for the Cu–13.5Al–4Ni SMA/CNT composites gradually decreased with the increase in the CNT content above 0.6 wt%. The Cu–13.5Al–4Ni–0.8CNT composite exhibits the smallest internal friction peak with tan δ of approximately 0.14. Figure 8 plots the tan δ values of the IF peaks of Cu–13.5Al–4Ni–*x*CNT (*x* = 0, 0.2, 0.4, 0.6, and 0.8 wt%) SMA/CNT composites determined from Fig. 7a as a function of CNT content. The tan δ values of the IF peaks of typical Cu–Al–Ni SMAs with various chemical compositions are also presented in Fig. 8 for comparison^36^. The damping capacity of a material refers to the dissipation of energy by the reversible microstructural movement such as movement of martensite variant interfaces and moveable twins in the martensite phase or irreversible thermoelastic process inside the materials during mechanical vibrations^42–44^. Besides, it has been reported that martensite morphology can also influence damping performance of SMAs^45^. This effect should be less irrelevant in this study because the Cu–13.5Al–4Ni–*x*CNT SMA/CNT composites all exhibited a similar martensite morphology, as shown in Figs. 3 and 4.

According to the Delorme equation, the damping capacity of the SMAs is proportional to the quantity of transformed martensite^46^:

1 $$\mathrm{tan}\delta \sim \frac{1}{\omega }.\frac{d\psi ({V}_{m})}{d{V}_{m}}.\frac{d{V}_{m}}{dT}.\frac{dT}{dt},$$

where ${V}_{m}$ is the volume fraction of the transformed martensite, *ω* is the angular frequency of the applied stress, $\psi \left({V}_{m}\right)$ is a monotonic function associated with the transformation volume change and/or shape strain, and $\frac{d\psi }{d{V}_{m}}$ is a constant for all thermoelastic martensite structures. Based on the measured transformation enthalpies (∆*H*), as shown in Fig. 2b, with the increase in the CNT content, the magnitude of the austenite-to-martensite transformation decreased. Therefore, the CNT-modified Cu–13.5Al–4Ni–*x*CNT composites with a lower ∆*H* exhibit lower austenite-to-martensite transformations, and therefore lower damping capacities. Besides, the Cu–13.5Al–4Ni–0.8CNT composite exhibited the lowest internal friction peak, which can also be attributed to the pinning effect of the finely dispersed precipitated ${\gamma }_{2}$ phase particles, which impeded the movements of the austenite/martensite interfaces and martensite variants in the matrix^10^.

As shown in Fig. 7b, the storage modulus curve of each Cu–13.5Al–4Ni–*x*CNT specimen reaches a minimum during the $\upbeta {(DO}_{3})$ → ${\upbeta }_{1}{\prime}\left(18\mathrm{R}\right)$ MT. After the $\upbeta {(DO}_{3})$ → ${\upbeta }_{1}{\prime}\left(18\mathrm{R}\right)$ MT, the storage modulus in ${\upbeta }_{1}{\prime}\left(18\mathrm{R}\right)$ martensite slightly increased with the decrease in temperature. The storage modulus minima of the Cu–13.5Al–4Ni–*x*CNT composites containing 0, 0.2, and 0.4-wt% CNTs are approximately 20,000 MPa during MT. Nevertheless, the storage modulus minimum of the Cu–13.5Al–4Ni–*x*CNT composite increases to approximately 35,000 and 42,000 MPa when the CNT content increases to 0.6 and 0.8 wt%, respectively. The storage modulus of a material is related to the ability of the material to store energy elastically during deformation. The loss modulus represents the amount of dissipation of energy because of the internal motions during deformation. Xue et al.^38^ reported that a small quantity of CNTs added in the Cu matrix can significantly improve the mechanical strength and Young’s modulus (modulus of elasticity) of the Cu–CNTs composites. Chawla et al.^47^ indicated that, in composites with low volume fractions of reinforcement, the sliding of dislocations has a key role in interpreting the deformation mechanism. Hence, the aggregation of CNTs prevented the dislocation sliding and led to piling of the dislocations at the interfacial area during the tensile loading. The pile of dislocations inside the crystalline lattice considerably reduced the ability of plastic deformation of the composites. In this study, the increment of the CNTs in the modified Cu–13.5Al–4Ni composite/SMAs hindered the dislocation and other internal motions inside the crystalline lattice and improved the deformation elastically. As the storage modulus is related to the energy storage elastically, the increase in the CNT content led to the increase in the storage modulus. Therefore, the Cu–13.5Al–4Ni–0.6CNT and Cu–13.5Al–4Ni–0.8CNT composites exhibited remarkably higher storage moduli than those of the Cu–13.5Al–4Ni–*x*CNT composites with CNT contents below 0.4 wt%.

The strengthening effect of the CNTs in the Cu–13.5Al–4Ni–0.6CNT and Cu–13.5Al–4Ni–0.8CNT composites could be attributed to the following mechanisms^48^: (1) load transfer strengthening (${\Delta \sigma }_{LT}$), (2) grain refinement strengthening (${\Delta \sigma }_{gb})$, and (3) Orowan strengthening (${\Delta \sigma }_{Or}$) and dislocation strengthening (${\Delta \sigma }_{Dis})$. The yield strength (${\sigma }_{ys})$ is governed by

2 $${\sigma }_{ys}={\sigma }_{0}+{\Delta \sigma }_{gb}+{\Delta \sigma }_{LT}+{\Delta \sigma }_{Dis}+{\Delta \sigma }_{Or}.$$

Carneiro et al.^48^ showed that, in Cu/CNT composites, the addition of CNTs did not significantly affect the Cu matrix’s dislocation density, grain size, and texture. The mechanism of load transfer strengthening has a leading role in interpreting the influence of CNTs on the strengthening of Cu/CNT composites. Duong et al.^49^ observed that, in Cu/CNT composites, the enhanced hardness and tensile strength are attributed to the interfacial bond strength between the strengthening materials (CNTs) and Cu matrix. Therefore, the increment in hardness with the CNT content is related to the participation of the more interfaces between the Cu matrix and CNTs, which act as a bridge to transfer the mechanical loads, and therefore increase the elastic modulus (${E}{\prime}$). Besides, the Cu–13.5Al–4Ni–0.8CNT composite exhibits the highest storage modulus minimum during MT, also corresponding to the abundant precipitated ${\gamma }_{2}$ phase particles in the matrix.

## Conclusions

In this study, we investigated the modification of the mechanical strength and internal friction of Cu–13.5Al–4Ni SMAs by CNTs. The following conclusions were obtained.

1. The addition of small amounts of CNTs into Cu–13.5Al–4Ni SMAs did not influence the microstructure and MT sequence of the alloys. Nevertheless, the peak temperatures and $\Delta H$ values of the martensitic transformation peak gradually decreased with the increase in the amount of CNT addition.
2. The addition of CNTs had opposite effects on the austenite–martensite transformation of the Cu–13.4Al–4Ni SMA. However, the addition of the 0.8-wt% CNT significantly increased the mechanical strength of the modified alloys.
3. The reduction in $\mathrm{tan}\delta$ of the internal friction for CNT contents above 0.6 wt% was related to the reduction in the magnitude of the austenite-to-martensite transformation and existence of precipitated particles of γ_2_ (Cu_9_Al_4_).
4. The small amount of CNTs (0.8 wt%) enhanced the hardness through the participation of the more interfacial bonds between CNTs and matrix, which act as a connection to transfer the mechanical loads, and therefore increase the modulus of elasticity.

## Data Availability

The datasets used and/or analyzed during the current study are available from the corresponding author on reasonable request.
